# Basophil Activation Test Utility as a Diagnostic Tool in LTP Allergy

**DOI:** 10.3390/ijms23094979

**Published:** 2022-04-29

**Authors:** José A. Cañas, Natalia Pérez-Sánchez, Leticia Lopera-Doblas, Francisca Palomares, Ana Molina, Joan Bartra, María J. Torres, Francisca Gómez, Cristobalina Mayorga

**Affiliations:** 1 Allergy Research Group, Instituto de Investigación Biomédica de Málaga-IBIMA, 29009 Malaga, Spain; joseantonio.canas@ibima.eu (J.A.C.); leticia.ld87@gmail.com (L.L.-D.); francis.palomares@gmail.com (F.P.); anamolina22@hotmail.com (A.M.); 2 Andalusian Centre for Nanomedicine and Biotechnology-BIONAND, 29590 Malaga, Spain; mjtorresj@uma.es; 3 Allergy Clinical Unit, Hospital Regional Universitario de Málaga, 29009 Malaga, Spain; natbel.ps@gmail.com (N.P.-S.); paquigomez.p@hotmail.com (F.G.); 4 Allergy Section, Pulmonology, Hospital Clinic, University of Barcelona, 08036 Barcelona, Spain; jbartra@clinic.cat; 5 Clinical & Experimental Respiratory Immunoallergy (IRCE), Instituto de Investigaciones Biomédicas Pi I Sunyer (IDIBAPS)-ARADyAL, 08036 Barcelona, Spain; 6 Medicine Department, University of Malaga-UMA, 29010 Malaga, Spain

**Keywords:** basophil activation test, diagnosis, flow cytometry, nonspecific lipid transfer proteins, Pru p 3

## Abstract

Plant-food allergy is an increasing problem, with nonspecific lipid transfer proteins (nsLTPs) triggering mild/severe reactions. Pru p 3 is the major sensitizer in LTP food allergy (FA). However, in vivo and in vitro diagnosis is hampered by the need for differentiating between asymptomatic sensitization and allergy with clinical relevance. The basophil activation test (BAT) is an ex vivo method able to identify specific IgE related to the allergic response. Thus, we aimed to establish the value of BAT in a precise diagnosis of LTP-allergic patients. Ninety-two individuals with peach allergy sensitized to LTP, Pru p 3, were finally included, and 40.2% of them had symptoms to peanut (*n* = 37). In addition, 16 healthy subjects were recruited. BAT was performed with Pru p 3 and Ara h 9 (peanut LTP) at seven ten-fold concentrations, and was evaluated by flow cytometry, measuring the percentage of CD63 (%CD63^+^) and CD203c (%CD203c^high^) cells, basophil allergen threshold sensitivity (CD-Sens), and area under the dose–response curve (AUC). Significant changes in BAT parameters (%CD63^+^ and %CD203c^high^) were found between the controls and patients. However, comparisons for %CD63^+^, %CD203c^high^, AUC, and CD-Sens showed similar levels among patients with different symptoms. An optimal cut-off was established from ROC curves, showing a significant positive percentage of BAT in patients compared to controls and great values of sensitivity (>87.5%) and specificity (>85%). In addition, BAT showed differences in LTP-allergic patients tolerant to peanut using its corresponding LTP, Ara h 9. BAT can be used as a potential diagnostic tool for identifying LTP allergy and for differentiating peanut tolerance, although neither reactivity nor sensitivity can distinguish the severity of the clinical symptoms.

## 1. Introduction

Food allergy (FA) is an adverse response of the immune system triggered by innocuous food-protein antigens. This pathological status results from exposure to allergenic foods, which causes mild/severe clinical symptoms such as gastrointestinal disorders, oral allergy syndrome (OAS), urticaria, angioedema, and, sometimes, anaphylaxis, which can be life-threatening [1]. Over recent years, FA has exponentially grown worldwide [2], reaching rates higher than 10% [3], and becoming a health burden. Moreover, the main problem is the lack of effective treatment.

Lipid transfer proteins (LTPs), from the fruits of several Rosaceae plants, are commonly responsible for allergic reactions. Unfortunately, LTPs are widespread in foods and it is hard to predict the possible reactions. In fact, Pru p 3, which belongs to the LTP family, is the major allergen of peach and the primary sensitizer in the Mediterranean population [4,5], although expanding to other geographical areas [6,7,8,9,10,11,12]. Moreover, other LTPs such as Ara h 9 produce peanut allergy [13,14].

Currently, one of the main problems for FA is achieving a precise diagnosis. Over recent years, expert boards and practice guidelines have established several diagnostic modalities, such as clinical history, physical examination, elimination diets, skin prick tests (SPTs), and specific IgE (sIgE) in vitro determination. However, they have several disadvantages, including controversial results with high sensitivity but low specificity [15]. To solve this, oral food challenge (OFC) is necessary for a correct diagnosis [16,17,18], although the occurrence of adverse reactions is sometimes life-threatening [15]. Thus, searching for new diagnostic methods is essential.

The basophil activation test (BAT) has emerged as an in vitro functional assay [19] for a precise diagnosis of allergic diseases, and some authors have indicated that it provides information about the severity of reactions, as it has been demonstrated for FA [20]. However, most studies with BAT in plant-food allergy are related to peanut but not to sensitization related to peach-derived LTPs [19,21]. BAT is based on the basophil response to allergens, measuring the levels of basophil activation markers (such as CD63 and CD203c) by flow cytometry [22,23,24]. BAT-derived results can be shown as basophil reactivity, which is the percentage of cells that express activation markers. Likewise, other ways of interpreting the results are the basophil allergen threshold sensitivity (CD-sens) and area under the dose–response curve (AUC), which are novel promising BAT-derived parameters to evaluate basophil reactivity and allergen sensitivity [25].

Although this test could be very useful for diagnosis, BAT-derived results should be carefully considered and must be complementary to other diagnostic methods such as clinical history, SPT, or in vitro sIgE determination. Despite their advantages, BAT has several limitations that must be overcome. These include standardization of the laboratory procedure and the flow cytometry data analyses in large prospective cohorts, to avoid false positives.

In this sense, our main objective is to study if BAT could be used as a diagnostic tool to distinguish controls from peach-allergic patients sensitized to LTPs. Furthermore, we evaluated if BAT could differentiate patients allergic or tolerant to peanut, and if it is related to the severity of the symptoms.

## 2. Results

### 2.1. Clinical and Demographic Parameters Evaluated in Study Population

**Figure 1:**
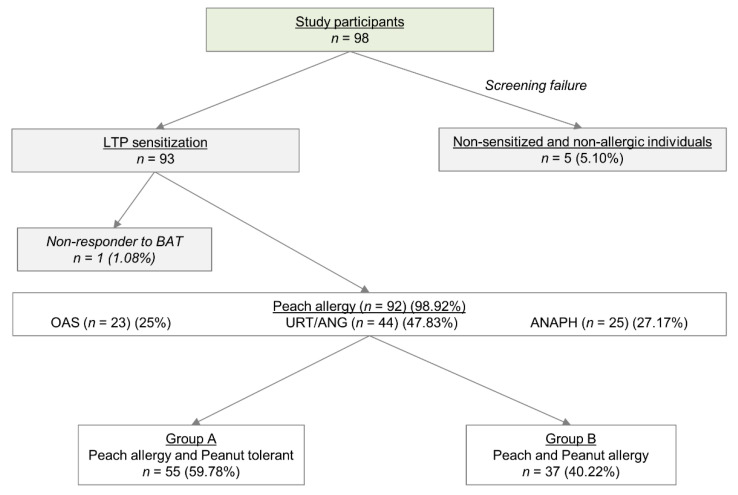
Flow chart of the patient classification. Nonresponder patients in BAT, and nonsensitized and nonallergic individuals were excluded from the study. The remaining patients were classified according to clinical history into three severity groups: oral allergy syndrome (OAS), urticaria/angioedema (URT/ANG), and anaphylaxis (ANAPH). Furthermore, these peach-allergic patients were divided into patients allergic to peach (Group A) and individuals allergic to peach and peanut (Group B). These groups of patients were used to compare them in clinical and BAT parameters terms.

In total, 98 subjects with a consistent history of peach allergy were initially enrolled. From these, 93 had a positive SPT to peach extract enriched with Pru p 3, and positive in vitro sIgE to Pru p 3. One patient was a nonresponder to BAT and was removed from the study (Figure 1). The final group of peach-allergic patients (*n* = 92) was analyzed. Most patients were female (68.48%) in the third decade of life (33.22 ± 10.22 years). Additionally, patients were categorized according to the clinical history and the severity of symptoms after peach intake such as: OAS (25%), urticaria/angioedema (URT/ANG) (47.83%), and anaphylaxis (ANAPH) (27.17%) (Figure 1). Moreover, 16 aged- and sex-matched control subjects without any plant food-allergy and confirmed tolerance particularly to peanut and peach were recruited. Clinical and demographic characteristics are shown in Table 1.

The wheal area in the SPT performed with peach extracts in the clinical groups tended to increase with the severity of symptoms (Table 1); a statistical increase was found in the SPT performed with peanut in patients allergic to both foods (*p* < 0.05; Table S1: Clinical and demographic characteristic of recruited individuals).

Moreover, we checked the sensitization to profilin in these groups and, interestingly, observed a drop in the percentage of positive patients to profilin in the SPT as the severity of reported symptoms increased, showing significant differences between the OAS and anaphylaxis group (*p* < 0.05, Table 1).

The results of the SPT to peach, peanut, and profilin were negative in all individuals included in the healthy control group.

Although not significantly different, serum in vitro total IgE (tIgE) was higher in patients than in controls (Table 1). Regarding serum in vitro sIgE to Pru p 3 and Ara h 9, levels were significantly higher in FA patients and in all clinical groups compared to healthy controls (*p* < 0.01–0.0001, Table 1). However, although in vitro sIgE to Pru p 3 showed a trend toward increasing with severity, and Ara h 9 in vitro sIgE appeared to be decreasing with the severity of allergic reactions, no significant differences were found (Table 1).

### 2.2. Determination of Optimal Doses for Maximal Basophil Response

**Figure 2:**
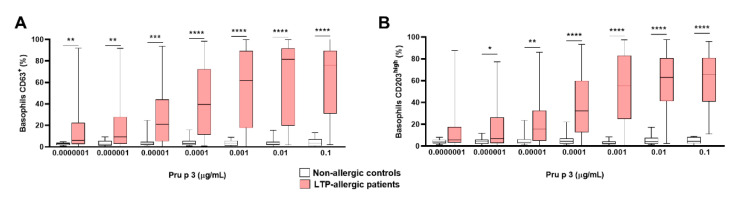
BAT performed with optimal doses of Pru p 3 differentiates between nonallergic controls and LTP-allergic patients. Dose–response curves were performed with seven ten-fold concentrations of Pru p 3 comparing the BAT parameters %CD63^+^ (**A**) and %CD203c^high^ (**B**). Almost all Pru p 3 concentrations showed significant differences in basophil activation terms for %CD63^+^ and %CD203c^high^ in patients compared to healthy controls. * *p* < 0.05, ** *p* < 0.01, *** *p* < 0.001, **** *p* < 0.0001.

First, it is important to establish the optimal protein concentration for maximal cellular and specific activation for each allergen, Pru p 3 [26]. Thus, we performed dose–response curves with 16 healthy controls and 92 LTP-allergic patients at seven Pru p 3 concentrations (from 0.1 to 0.0000001 μg/mL) to challenge the basophils in vitro. The expression of basophil-specific CD63 was significantly increased in LTP-allergic patients versus controls in a dose-dependent manner of Pru p 3 from 0.0000001 μg/mL up to 0.001 μg/mL followed by a plateau (Figure 2A). Likewise, similar behavior was observed in the expression of CD203c (Figure 2B). We detected that the optimal discriminating concentrations of Pru p 3 were the three highest (0.001, 0.01, and 0.1 μg/mL), which were selected for further analysis.

### 2.3. BAT Differentiates LTP-Allergic Patients from Controls, but It Does Not Distinguish Severity Clinical Groups in These Patients

**Figure 3:**
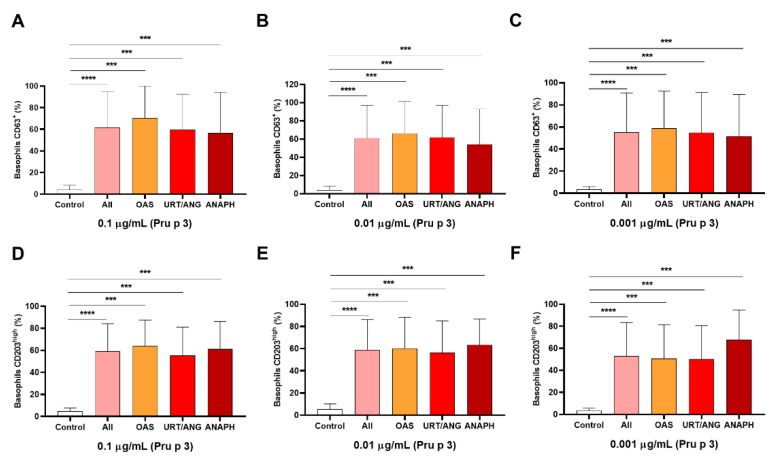
BAT does not distinguish LTP-allergic patients according to severity of symptoms. BAT parameters %CD63^+^ (**A**–**C**) and %CD203c^high^ (**D**–**F**) were compared between healthy controls, the whole LTP-allergic population, and the different clinical severity groups at the three selected concentrations of Pru p 3. In all cases, significantly higher activations of basophils, measured in terms of %CD63^+^ and %CD203c^high^, were found between all LTP-allergic patients and controls, and the different clinical groups (OAS, URT/ANG, and ANAPH) and healthy individuals. Levels of %CD63^+^ and %CD203c^high^ were similar among patients with OAS, URT/ANG, and ANAPH in all concentrations of Pru p 3 used. *** *p* < 0.001, **** *p* < 0.0001. ANAPH, anaphylaxis; OAS, oral allergy syndrome; URT/ANG, urticaria/angioedema.

Then, we tested whether BAT parameters could differentiate among severity groups (OAS, URT/ANG, and ANAPH). We observed that %CD63^+^ values were significantly higher in patients (*p* < 0.0001) and in all different severity groups (*p* < 0.001), compared to controls in the three concentrations of Pru p 3 established (Figure 3A–C). This pattern was similar to %CD203c^high^ values (Figure 3D–F). However, these BAT parameters did not differentiate among the clinical groups (Figure 3A–F).

**Figure 4:**
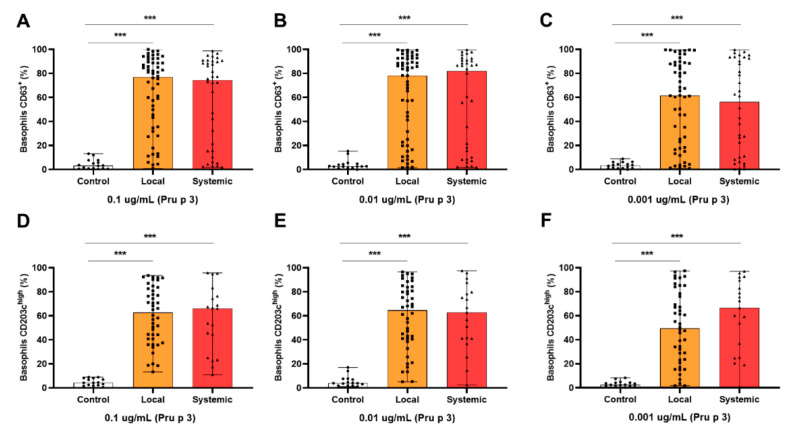
BAT parameters did not differentiate patients grouped according to symptoms’ location. Comparisons of BAT parameters %CD63^+^ (**A**–**C**) and %CD203c^high^ (**D**–**F**) were also made among controls and patients with local or systemic symptoms at the three selected concentrations of Pru p 3 (0.1, 0.01, and 0.001 µg/mL). BAT parameters %CD63^+^ and %CD203c^high^ were significantly higher between patients with local or systemic symptoms in comparison to healthy controls. However, no changes were found in basophil activation between LTP-allergic individuals with local or systemic reactions. *** *p* < 0.001.

Likewise, patients were grouped depending on if they developed local or systemic symptoms. Similarly to previous results, %CD63^+^ and %CD203c^high^ levels did not change between groups, although both showed significantly higher levels than those of the controls (*p* < 0.001; Figure 4).

Furthermore, we performed correlation analysis between wheal area in the SPT vs. severity or percentage of activation of both %CD63^+^ and %CD203c^high^ parameters. In none of the cases were any significant correlation observed (data not shown), showing that SPT to LTP does not correlate with severity, as it happens in BAT.

**Figure 5:**
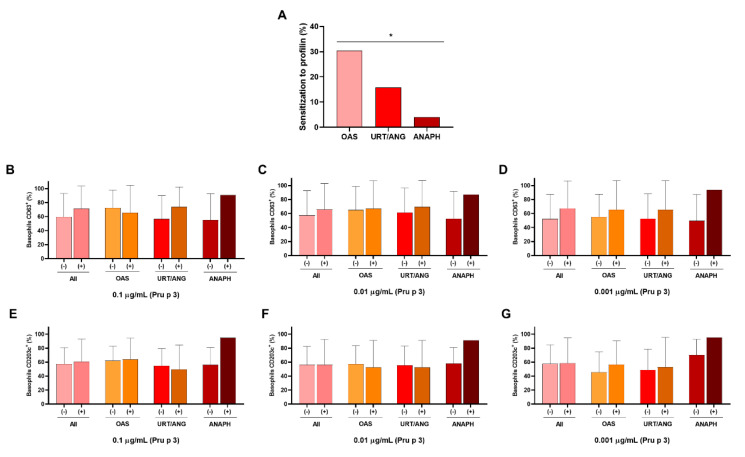
Sensitization to profilin ameliorates the severity of symptoms from LTP-allergic patients. (**A**) The percentage of sensitized patients to profilin was determined, showing a decrease in the number of LTP-allergic patients with sensitization to profilin in relation to the increase in severity of symptoms. Moreover, levels of %CD63^+^ (**B**–**D**) and %CD203c^high^ (**E**–**G**) were compared between LTP-allergic patients sensitized or not to profilin at the three selected concentrations of Pru p 3, not showing changes in any case. * *p* < 0.05. ANAPH, anaphylaxis; OAS, oral allergy syndrome; URT/ANG, urticaria/angioedema.

Taking into account that profilin could be a confounding factor in relation to the severity of the clinical symptoms [27], we analyzed its influence on our patient population. As previously found, we confirmed that patients with profilin sensitizations were those with weak or moderate symptoms, whereas patients not sensitized to profilin showed more severe symptoms such as anaphylaxis (Figure 5A). When analyzing the basophil reactivity in patients depending on their sensitization to profilin, we did not find any significant differences for both%CD63^+^ and %CD203c^high^ and the different clinical symptoms (Figure 5B–G). Likewise, we did not observe any differences when the wheal area of the SPT of both peach and peanut was compared among all severity groups (data not shown).

### 2.4. Other Ways of Interpreting BAT in LTP-Allergic Patients

**Figure 6:**
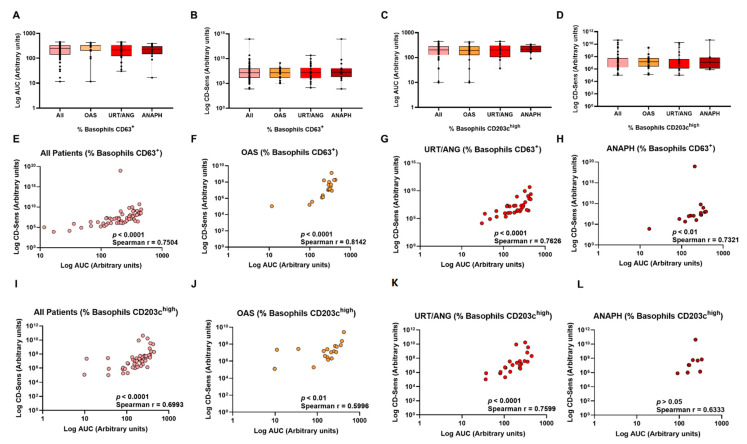
AUC and CD-Sens parameters do not distinguish clinical severity groups. (**A**–**D**) Comparisons of values of AUC and CD-Sens obtained from %CD63^+^ and %CD203c^high^ parameters. No differences were found in these BAT parameters among compared groups. Correlations between AUC and CD-Sens from %CD63^+^ (**E**–**H**) and %CD203c^high^ (**I**–**L**) were performed, showing positive significant correlations in the clinical groups. ANAPH, anaphylaxis; OAS, oral allergy syndrome; URT/ANG, urticaria/angioedema.

To assess whether basophil sensitivity could differentiate the different clinical groups, we decided to analyze CD-sens and AUC [21,28,29,30], and we observed similar levels among the different severity groups (Figure 6A–D). When these parameters were analyzed independently in each severity group and for each BAT parameter (%CD63^+^ and %CD203c^high^), we found that although strong positive correlations between CD-sens and AUC were found (r > 0.7, *p* < 0.01–0.0001), except for the anaphylaxis group in %CD203c^high^ parameters (Figure 6E–M), AUC and CD-Sens parameters did not distinguish clinical severity groups.

### 2.5. Principal Component Analysis Differentiates LTP-Allergic Patients from Controls but It Does Not Distinguish Severity Clinical Groups

**Figure 7:**
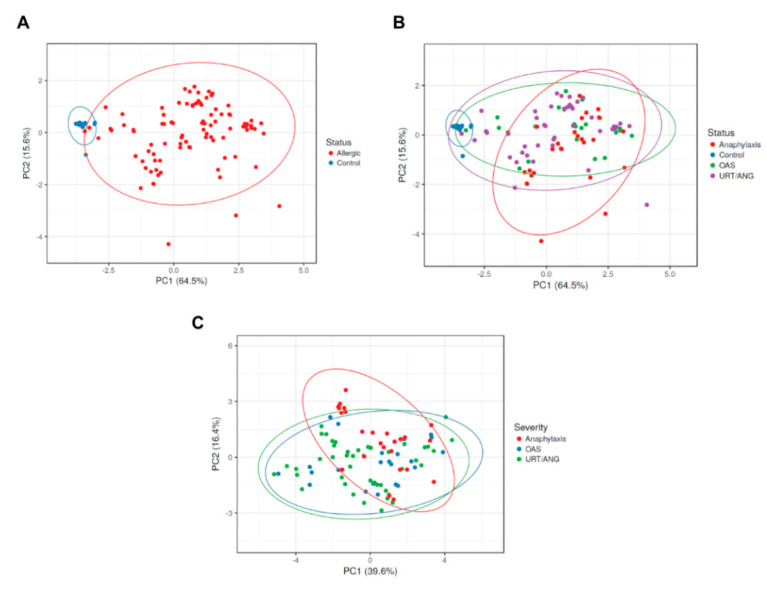
Principal component analysis is able to differentiate control subjects from peach-allergic patients, but not among patients grouped by symptoms. Using in vitro sIgE to Pru p 3, %CD63^+^, and %CD203c^high^ as variables to perform PCA, healthy controls were well differentiated from allergic patients (**A**). However, this fact did not occur among the severity groups (**B**), where more homogeneity was observed. (**C**) No separated clusters were obtained in the PCA performed with the variables in vitro sIgE to Pru p 3, wheal area, AUC, CD-sens, %CD63^+^ and %CD203c^high^, when severity groups were analyzed. OAS, oral allergy syndrome; PC, principal component; URT/ANG, urticaria/angioedema.

To confirm these results, we performed a principal component analysis (PCA) to visualize the variability of data (Figure 7). First, we performed a PCA using in vitro sIgE to Pru p 3, %CD63^+^, and %CD203c^high^ as variables with both healthy controls and allergic populations (Figure 7A), and then in healthy controls and allergic patients with different clinical severity (Figure 7B). It allowed differentiating the control group from allergic patients, and the two principal components explained the 80.1% of variability obtained (Figure 7A). Controls were also discriminated from severity groups (Figure 7B). However, when PCA was performed in the groups of different severity with the variables in vitro sIgE to Pru p 3, SPT wheal area, AUC, CD-sens, %CD63^+^, and %CD203c^high^, no clusters were found, and the explained variability by the two principal components was 56% (Figure 7C).

These data could indicate that the allergic population of this study is homogeneous regarding in vivo and vitro variables, in spite of the different symptoms suffered.

### 2.6. BAT to Pru p 3 Could Be Used as Supporting Diagnostic Tool to Distinguish Peach-Allergic Subjects and Controls

**Figure 8:**
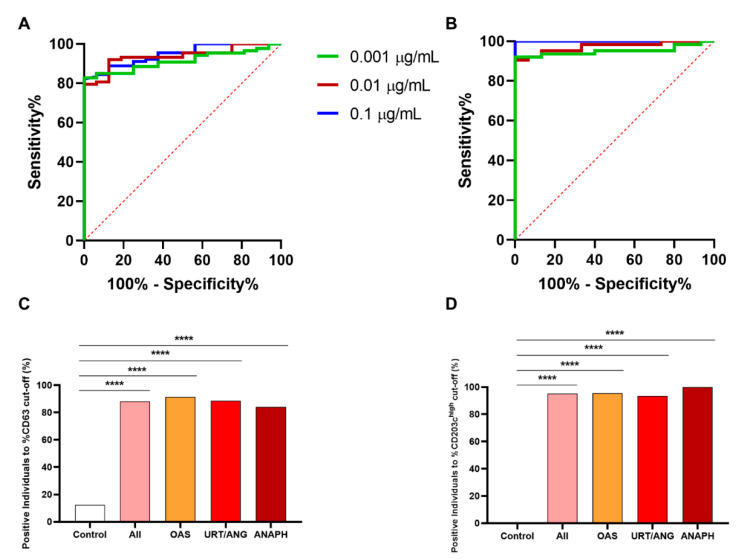
ROC curve analysis for BAT in predicting peach allergy. ROC curves of %CD63^+^ (**A**) and %CD203c^high^ (**B**) were performed at the three selected concentrations of Pru p 3 (0.1, 0.01, and 0.001 µg/mL). The positivity of patients to BAT were calculated using optimal cut-off values from %CD63^+^ (**C**) and %CD203c^high^ (**D**), not showing any differences between the severity group, but these groups and the whole allergic patients had a significantly higher percentage of positivity in comparison to healthy controls. **** *p* < 0.0001. ANAPH, anaphylaxis; OAS, oral allergy syndrome; URT/ANG, urticaria/angioedema.

Receiver operating characteristic (ROC) curves were used to check the performance of BAT parameters (percentage of basophils CD63^+^ or CD203c^high^) in the diagnosis of peach allergy (Figure 8A,B). In both cases and with the three highest Pru p 3 concentrations selected as optimal, the AUC was higher than 0.90 and the specificity was higher than 87%, with high positive predictive (PPV) and negative predictive values (NPV) (Table 2).

Using the cut-off values shown in Table 2, we established the percentage of positivity in order to check if BAT distinguishes and categorizes controls and allergic patients. Positivity was determined as a value of %CD63^+^ or %CD203c^high^ higher than the cut-off value in at least two consecutive concentrations. Regarding the use of %CD63^+^ values, a positivity of 88% was found in the whole allergic population (Figure 8C). Likewise, in the different clinical groups, the positivity found was higher than 80%, showing significant differences with the control group (*p* < 0.0001, Figure 8C). On the other hand, using %CD203c^high^ values, the positivity in the whole allergic population and in the different severity groups was higher than 90%, exhibiting statistical differences compared to controls (*p* < 0.0001, Figure 8D). Percentages of positive individuals to BAT were similar among the whole allergic population and the different clinical groups for both %CD63^+^ and %CD203c^high^.

### 2.7. Differences in Reactivity to LTPs, Pru p 3 or Ara h 9, Depending on Peanut Tolerance

Patients with peach allergy were further classified in relation to their additional allergy or tolerance to peanut, and we observed that 55 (59.78%) patients were tolerant to peanut (Group A), from which 54 (98.18%) had a positive SPT for peach and 6 for peanut (10.90%), and 12 (21.82%) were peach-allergic confirmed by OFC and consumed peanuts in their regular diet regardless of SPT results. Thirty-seven patients (40.22%) were also allergic to peanut in addition to presenting peach allergy (Group B) (Figure 1). From these, 31 (83.78%) had a positive SPT for peanut, and six (16.22%) were peanut-allergic confirmed by OFC. To rule out sensitization to storage proteins in Group B, all patients were tested for in vitro sIgE at Ara h 2, and all were negative (data not shown). Likewise, Pru p 3 and Ara h 9 in vitro sIgE levels were significantly higher in the peach- and peanut-allergic group (Group B) compared to patients allergic only to peach (Group A) (Table S1: Clinical and demographic characteristic of recruited individuals).

We afterward evaluated responses in BAT to different nsLTP, Pru p 3, and Ara h 9, in Group A and Group B.

First, we performed multiple comparisons regarding BAT parameters, for Pru p 3 and Ara h 9, among controls, the whole allergic population, Group A, and Group B. In all cases, we observed significant differences between controls and the rest of groups (*p* < 0.001–0.0001), but there were no differences in BAT between mono- (Group A) or poli-sensitized (Group B) patients to Pru p 3 and/or Ara h 9 (data not shown).

**Figure 9:**
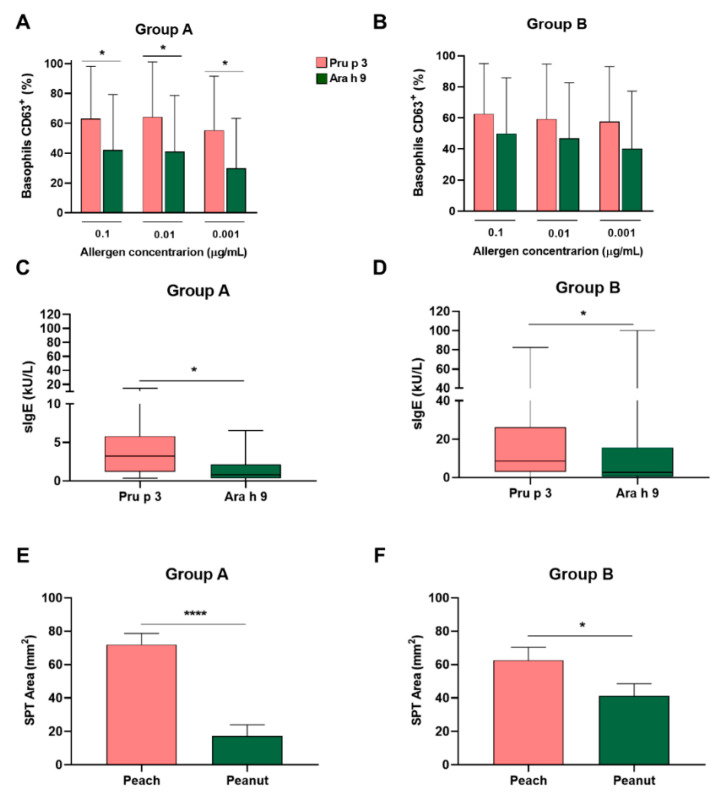
BAT is able to differentiate peanut tolerance. Percentage of basophils CD63^+^ was compared between BAT performed with Pru p 3 and Ara h 9 in patients with peach allergy and peanut tolerance (Group A) (**A**) and in individuals allergic to peach and peanut (Group B) (**B**), showing higher levels when BAT was made with Pru p 3. In addition, levels of in vitro sIgE to Pru p 3 and Ara h 9 were compared in Group A (**C**) and Group B (**D**), showing significantly higher levels of sIgE to Pru p 3 in both groups. Likewise, the wheal area from SPT performed with peach enriched with Pru p 3 and peanut extracts was compared in Group A (**E**) and in Group B (**F**), showing a higher area of wheal to peach extract in both groups. * *p* < 0.05; **** *p* < 0.0001.

Secondly, when BAT parameters from Group A individuals were evaluated, the results showed that %CD63^+^ values were significantly higher in BAT performed with Pru p 3 compared to Ara h 9 in all concentrations tested (*p* < 0.05; Figure 9A), but not with %CD203c^high^ values (data not shown). Interestingly, when analyzing individuals from Group B, we found similar levels of %CD63^+^ in BAT performed with Pru p 3 or Ara h 9 (Figure 9B) and for %CD203c^high^ (data not shown). Of note, the in vitro sIgE and SPT area were significantly higher to Pru p 3 compared to Ara h 9 not only in allergic- and peanut-tolerant subjects (Group A), but also in peach- and peanut-allergic patients (Group B) (*p* < 0.05; Figure 9C–F).

All these data could indicate that BAT, using CD63 as an activation marker, shows a lower reactivity to its corresponding LTP, Ara h 9, in peanut-tolerant patients.

## 3. Discussion

This study demonstrates that basophil activation to Pru p 3 is useful to distinguish peach-allergic patients sensitized to LTPs and subjects tolerating this food, as well as to identify the primary sensitization to this allergen; but it cannot predict the severity of reactions to peach that could be rather related to profilin sensitization. LTP allergy, and specifically peach allergy, is one of the most common fruit allergies in the adolescent and adult population of southern Europe, and may account for up to 96% of FA cases [31,32]. Peach-allergic patients suffer from immediate immune reactions after peach intake; however, they can also develop more severe symptoms, even life-threatening reactions as it has been described in southern Europe and, particularly, Spain [4]. In this study, we observed that 25% experienced OAS, 47.83% angioedema/urticaria, and 27.17% anaphylaxis. Therefore, a search of novel and effective diagnostic methods is necessary.

Out of all the available techniques, BAT using flow cytometry has emerged as a powerful tool to detect several allergies [20]; indeed, the benefits of BAT in other FAs have already been demonstrated [33]. It detects sensitization on basophils indirectly, and could be used if conventional tests are negative, not available, or if the performance of an OFC could be potentially life-threatening. Allergen incubation in whole blood samples produces basophil degranulation in sensitized individuals and the overexpression of activation markers such as CD63 and, occasionally, CD203c.

We demonstrated that BAT distinguishes peach-allergic patients sensitized to LTPs from controls. However, these parameters (%CD63^+^, %CD203c^high^) cannot differentiate clinical groups, which limits its utility-predicting severity. Although there are few studies on BAT in LTP-associated peach allergy, the clinical utility of BAT in the diagnosis and prediction of severity of mugwort pollen-related peach allergy has been recently demonstrated [34]. Moreover, other studies also showed the utility of BAT in FA diagnosis, discriminating peanut-tolerant and -allergic children [19,21]. These discrepant results with ours could be explained by the different allergen sensitization profile and the allergological work-up made for diagnosing patients. The diagnostic work-up could be based on clinical history and the symptoms referred by patients that cannot be accurate enough, limiting the clinical classification precision [34,35]. Moreover, OFC, although potentially life-threatening, may be more useful for confirming the diagnosis. However, OFC can be a limited tool for classifying the patients according to the severity because, following the protocol, the procedure could be stopped as symptoms appear, in order to confirm allergy reaction and minimize the risk of severe reaction. Therefore, the definitive clinical symptoms could not be achieved, for ethical reasons. Moreover, there are other factors that may not be reproduced during OFC, such as the exact amount of LTP that caused the reaction in the patient, or other patient-specific factors that may influence the severity of the reaction [36]. Therefore, although OFC remains the gold standard for diagnosis, it is not always possible to reproduce exactly the reaction the patient had. Co-sensitization with other pan-allergens, such as profilin, could affect basophil reactivity. Therefore, the percentage of activated basophils in the different severity groups could be similar regarding reactivity to LTPs but different according to profilins, and this could be the reasons why BAT parameters are similar in all clinical groups.

In addition to this, it has been reported that profilin could play a role of confounding factors interfering with the severity of the symptoms [37]. In fact, in our previous study, it has been demonstrated that co-sensitization to profilin is causative for less severe reactions in response to LTPs [27]. One possible reason why profilin exerts a protective role in LTP allergy could be the different affinity in binding to IgE of LTP and profilin pan-allergens. Although more studies are necessary to elucidate this phenomenon, we could hypothesize that profilin and nsLTP allergens compete in their binding to sIgE sites. The phenomenon of the protective role of profilin has also been confirmed in the current study; however, we observed that this fact does not affect basophil or mast cell reactivity to LTPs since neither BAT nor SPT results are modified with the profilin sensitization. Nevertheless, further studies are necessary in order to analyze the role of BAT reactivity to profilin in patients with different severity grades.

For interpreting BAT results, the AUC and the dose–response curve CD-sens have emerged as alternative ways to evaluate basophil reactivity and basophil sensitivity, respectively [21,26], although they are less used because they have several limitations [25]. One of them is that they cannot be calculated in nonallergic individuals; therefore, no comparisons between healthy controls and allergic patients can be performed. Moreover, CD-sens has to be measured with a dose–response curve, as the lowest allergen concentration gives 50% of the maximum upregulation of CD63 [25], and, in some cases, it is not possible to obtain. Although analyses of CD-sens have shown promising results predicting allergic reactions, few or no studies have been developed in peach allergy [38,39]. In contrast to these studies, we have not found any changes in AUC and CD-sens among clinical severity groups, which showed similar levels. Some authors have observed differences in CD-sens between patients with positive or negative food challenge results [38]; but other works have not found differences in CD-sens between children with and without OAS to a peanut challenge [25]. All these data should be interpreted with caution and more studies about CD-sens in LTP allergy must be performed to elucidate this question.

In the diagnosis of FA caused by LTPs from fruits, SPT with total extract is often used. However, it has a low specificity and allergenic potency [40]. Moreover, sIgE determination in vitro with whole extracts, although very sensitive, has low specificity due to the content of glycoproteins with IgE-binding capacity and to the presence of cross-reacting proteins [41]. BAT has been postulated to have high specificity and PPV and be an important addition to existing diagnostic tests, such as SPT and sIgE, which is very sensitivity but not specific [23]. The specificity and sensitivity of BAT for FA diagnosis have been described to be between 62–90% and 80–100%, depending on the allergen [20]. In our study, after determining the cut-off in ROC curves with several Pru p 3 concentrations and data of %CD63^+^ or %CD203c^high^, we observed a sensitivity between 80 and 100% and specificity values between 87.5 and 100%, as well as PPV and NPV higher than 95%, which can distinguish controls from peach-allergic patients with a high rate of BAT positivity (higher than 80%), and a low rate of false positives.

We have also studied the association of BAT with other clinical results for a diagnostic test, such as serum in vitro sIgE to Pru p 3, serum tIgE, and wheal area from the SPT. No significant correlations and associations among BAT parameters (%CD63^+^ or %CD203c^high^) and clinical results were found (data not shown). Some studies have demonstrated correlations between %CD63^+^ basophils and in vitro sIgE for Pru p 3 in peach allergy, and in other FA (egg and cow’s milk) [34,42], although the correlations were mild-moderate.

Nonspecific LTPs, such as Ara h 9 and Pru p 3, are ubiquitous panallergens that cause FA in adults in the Mediterranean areas [43]. In the studied population, we observed differential levels of %CD63^+^ in BAT performed with Pru p 3 and Ara h 9 in peach-allergic patients depending on peanut reactivity [44]. Nonspecific LTPs constitute relevant allergens in peanut and peach allergies, showing that Pru p 3 can be the primary sensitizer in the Spanish population [45]. When we analyzed the reactivity to both LTPs, by in vitro sIgE determination or SPT area, no differences in tolerance were found. Interestingly, a higher reactivity to Pru p 3 compared to Ara h 9 was found in BAT in patients who only react to peach and show tolerance to peanut, whereas a similar recognition of both Pru p 3 and Ara h 9 was observed in BAT in patients who exhibit clinical symptoms to both peach and peanut. Despite these interesting results, further studies including a larger population of peach-only-allergic patients could elucidate its value as a tool for taking OFC decisions in LTP patients.

We have found promising results about the use of BAT as a diagnostic tool. Nevertheless, these results must be confirmed in further studies in other larger and independent populations, with increased sample size in each severity clinical group. Finally, it must be taken into account that the diagnosis was based on clinical history, SPT, and/or sIgE, rather than on OFC, although it also has limitations in relation to severity definition. Therefore, due to these limitations, further studies are necessary.

## 4. Materials and Methods

### 4.1. Study Population

Adult subjects with a consistent history of peach allergy and positive SPT to peach extract enriched with Pru p 3, and positive in vitro sIgE to Pru p 3 were included. The classification of patients was performed according to clinical history into three severity groups: OAS, URT/ANG, and ANAPH, according to similar classifications proposed by other authors [46]. Additionally, patients were separated into two groups according to peanut tolerance: Group A (patients with confirmed tolerance to peanut independently of SPT/in vitro sIgE to peanut results); Group B (patients with consistent history to peanut allergy, positive SPT or OFC to peanut, and negative in vitro sIgE to Ara h 2). In addition, control subjects without plant-food sensitization or allergy were also included. The study was conducted according to the principles of the Declaration of Helsinki and approved by the Local Ethics Committee of Málaga (FIM-PRU-2018-01), Spain. All participants signed the informed consent.

### 4.2. Blood Collection

Blood samples were obtained in lithium heparin coagulant tubes (Vacuette^®^, Greiner Bio-One International GmbH, Kremsmünster, Austria) for BAT performance, and in anticoagulant-free tubes (BDVacutainer, Franklin Lakes, NJ, USA) for obtaining serum. In all cases, blood collection for BAT was prior to OFC.

The SPTs were performed using standardized peach-protein extracts enriched in Pru p 3, and peanut (ALK Abello, Madrid, Spain). In addition, the SPT to profilin was performed. Wheals of 7 mm^2^ in area were considered positive [47].

Levels of in vitro tIgE and sIgE against Pru p 3 and Ara h 9 were obtained by ImmunoCAP according to manufacturer’s indications (Thermo Fisher Scientific, Uppsala, Sweden).

### 4.3. Oral Food Challenge

A double-blind, placebo-controlled food challenge (DBPCFC) was performed following the European Academy of Allergy and Clinical Immunology (EAACI) recommendations [17]. The challenge procedure was performed in a hospital setting, with a specifically trained nurse and physician. Blinded active and placebo meals were randomly administered on separate days, and prepared immediately before the challenge. An external clinical investigator made the recipe and controlled the blinding for DBPCFC. In the active preparation, 200 mL of peach juice (quantified in 6.2 mg of Pru p 3) were masked within 50 mL of orange beverage (Sunny Delight Beverages Co., Barcelona, Spain) and red colorant in a final volume of 250 mL. In the placebo preparation, peach was replaced by 250 mL of orange beverage, 2 tablespoons of food thickener, and 4 drops of peach concentrate flavor and red colorant. Up to six doses were administered with a starting dose of 5 mL (31 mg of Pru p 3), increasing at 20 min intervals until reaching a total cumulative dose of 250 mL. The starting dose contained 31 mg of Pru p 3, and it was increased until reaching the highest dose of 1550 mg, corresponding to 120 g of pit-less unpeeled peach (about 1 peach of an average size) [48].

For peanut (dry-roasted peanut) DBPCFC, 15.5 units of peanuts (corresponding to 14 g of peanuts) were crushed and masked with bitter orange jam. Up to five doses were administered, starting with half a peanut (0.5 g), and increasing at 20 min intervals until reaching a total cumulative dose of 14 g.

After each dose, patients assessed the intensity of OAS on a 0–10 visual analog scale (VAS). Challenges were stopped at the first objective reaction or after three consecutive doses with OAS with VAS score > 2. The reaction was classified as local (symptoms restricted to the skin or mucosal sites of direct contact with the allergen, such as OAS or isolated digestive complaints) or systemic (involving organs far from the site of initial contact with the allergen, requiring absorption and dissemination, i.e., urticaria, anaphylaxis). After the last dose, the patient remained under observation for at least 2 h.

### 4.4. Basophil Activation Test

BAT was performed with heparinized peripheral blood against natural Pru p 3 (Roxall, Bilbao, Spain) and Ara h 9 (Indoor Biotechnologies, Cardiff, United Kingdom) at seven serial concentrations from 0.0000001 to 0.1 μg/mL. Basophils were analyzed by flow cytometry as described previously [49]. Moreover, AUC was used as a combination of basophil reactivity and sensitivity [28].

### 4.5. Statistical Analysis

Parametric data were expressed as mean ± standard deviation (SD), or median and Q1-Q3 for nonparametric data. Data were compared using the unpaired *t* test, Mann–Whitney test, and ANOVA with the Bonferroni post hoc test or Kruskal–Wallis test with Dunn’s post hoc test. Correlations were estimated by Spearman’s or Pearson’s correlations.

Optimal cut-off values for BAT, sensitivity and specificity, PPV, and NPV were calculated by ROC curve analysis.

Statistical analyses were performed using GraphPad Prism 8 (GraphPad Software Inc., San Diego, CA, USA).

Multivariate analyses of BAT results and clinical data were carried out with the ClustVis bioinformatic tool [50], by PCA.

## 5. Conclusions

This is one of the few studies evaluating the results of BAT in LTP allergy. We show that BAT parameters are useful to distinguish LTP-allergic patients from tolerant controls and its utility to distinguish tolerant patients to peanut using its specific LTP. However, it is not able to differentiate clinical groups according to the severity of symptoms after allergen ingestion that seems to be related to profilin sensitization.

## Figures and Tables

**Table 1:** Clinical and demographic characteristic of study population and clinical groups.

	Healthy Controls (*n* = 16)	Peach Allergic Patients to LTP Sensitization				
		All (*n* = 92)	OAS (*n* = 23)	URT/ANG (*n* = 44)	ANAPH (*n* = 25)	*p*-Value
Age (years) ^a^	34.25 ± 13.79	33.22 ± 10.22	32.30 ± 9.09	34.75 ± 11.10	31.22 ± 9.47	N.S.
Female (%)	68.75	68.48	56.52	72.73	72	N.S.
SPT peach enriched with Pru p 3 (mm^2^) ^b^	<7	52.0 (35.0–80.75)	36.0 (30.0–76.0)	54.0 (40.0–100.0)	65.0 (31.5–93.5)	N.S.
SPT peanut (mm^2^) ^b^	<7	40.0 (25.0–45.0)	25.0 (25.0–150.0)	30.0 (25.0–75.0)	30.0 (25.0–67.0)	N.S.
Profilin (%)	Negative	16.30	30.44	15.91	4.00	*
Total IgE (kU/L) ^b^	88.20 (42.70–150.0)	127.0 (62.0–352.0)	186.5 (98.5–239.3)	115.0 (59.0–368.0)	121.5 (59.33–627.5)	N.S.
Specific IgE (Pru p 3) (kU/L) ^b^	0.01 (0.003–0.13)	6.01 (2.34–13.2)	4.26 (1.73–7.71)	4.82 (2.0–13.18)	8.73 (2.95–19.95)	**/***/****
Specific IgE (Ara h 9) (kU/L) ^b^	0.004 (0.00025–0.085)	2.61 (0.35–20.40)	12.80 (2.02–38.9)	1.31 (0.0–2.61)	0.74 (0.26–5.69)	†

^a^ Mean ± SD; ^b^ Median (Q1-Q3); * *p* < 0.05 (OAS vs. Anaphylaxis); ** *p* < 0.01 (Controls vs. OAS); *** *p* < 0.001 (Controls vs. URT/ANG, and Controls vs. Anaphylaxis); **** *p* < 0.0001 (Controls vs. All); † *p* < 0.01 (Controls vs. All). N.S., not significant; OAS, Oral allergy syndrome; URT/ANG, angioedema/urticaria; ANAPH, anaphylaxis.

**Table 2:** ROC curve parameters of % CD63^+^ and % CD203c^high^ from Pru p 3.

	AUC	Cut-off	Sensitivity (%)	Specificity (%)	PPV (%)	NPV (%)	*p*-Value
%CD63^+^							
0.1 μg/mL	0.947	12.35	85.26	93.75	100	100	****
0.01 μg/mL	0.938	5.87	92.05	87.50	97.8	100	****
0.001 μg/mL	0.914	6.605	85.06	93.75	98.7	53.6	****
%CD203c^high^							
0.1 μg/mL	1	9.96	100	100	100	100	****
0.01 μg/mL	0.973	17.25	80.48	100	100	71.4	****
0.001 μg/mL	0.951	9.655	92.06	100	100	75.0	****

**** *p* < 0.0001; NPV, negative predictive value; PPV, positive predictive value.

## Data Availability

Not applicable.

## Supplementary Materials

The following supporting information can be downloaded at: https://www.mdpi.com/article/10.3390/ijms23094979/s1.
