# Supplementary material for: Basophil Activation Test Utility as a Diagnostic Tool in LTP Allergy
Source: Int J Mol Sci. 2022 Apr 29;23(9):4979. doi: 10.3390/ijms23094979 (PMC9105056; doi:10.3390/ijms23094979)
Supplement: Supplementary file 1 [file ijms-23-04979-s001.zip › ijms-1664976-supplementary.pdf]

Table S1. Clinical and demographic characteristic of recruited individuals.

	**Healthy controls (n=16)**	**Peach allergic patients to LTP sensitization**			
		**All (n = 92)**	**Group A (n = 55)**	**Group B (n = 37)**	***p*-value**
Age (years)[a]	34.25 ± 13.79	33.22 ± 10.22	33.84 ± 10.13	32.26 ± 10.44	N.S.
Female (%)	68.75	68.48	73.36	56.76	N.S.
SPT Peach enriched with Pru p 3 (mm^2)[b]	NA	52.0 (35.0- 80.75)	68.0 (36.0-83.25)	45.0 (25.0- 87.5)	N.S.
SPT Peanut (mm^2)[b]	NA	40.0 (25.0- 45.0)	0.0 (0.0- 25.0)	27.5 (25.0- 45.0)	†
Total IgE (kU/L)[b]	88.20 (42.70- 150.0)	127.0 (62.0-352.0)	112.0 (58.0- 214.0)	195.5 (61.58- 612.30)	N.S.
Specific IgE (Pru p 3) (kU/L)[b]	0.01 (0.003- 0.13)	6.01 (2.34- 13.2)	4.39 (1.52- 8.70)	8.82 (2.95-26.40)	‡/ § / ¶/#
Specific IgE (Ara h 9) (kU/L)[b]	0.004 (0.00025-0.085)	2.61 (0.35-20.40)	0.69 (0.35- 1.76)	2.61 (0.35- 20.40)	*/**/***

[a]Mean ± SD; [b]Median (IQR); † $p < 0.05$ (Group A vs. Group B); ‡ $p < 0.0001$ (Controls vs. All); § $p < 0.0001$ (Controls vs. Group A); ¶ $p < 0.0001$ (Controls vs. Group b); # $p < 0.01$ (Group A vs. Group B); * $p < 0.01$ (Controls vs. All); ** $p < 0.01$ (Controls vs. Group A); *** $p < 0.01$ (Controls vs. Group B). NA, not available; N.S., not significant; SPT, skin prick test. Group A: patients with confirmed peach allergy and tolerance to peanut; Group B: patients with consistent history to peach and peanut allergy.
